# Immune reconstitution inflammatory syndrome (IRIS) in HIV positive patients initiated on antiretroviral therapy (ART)

**DOI:** 10.1186/1742-4690-9-S1-P21

**Published:** 2012-05-25

**Authors:** Basavaprabhu Achappa, Deepak Madi Unnikrishnan, B Anand Venugopal

**Affiliations:** 1 Kasturba Medical College Hospital, Mangalore, India

## Background

IRIS is defined as ‘occurrence or manifestation of new opportunistic infections or existing opportunistic infections within six weeks to six months after initiating antiretroviral therapy with increase in CD4 count’. The objective of this study was to determine profile of IRIS in HIV Positive patients after initiation of ART and correlation of IRIS with CD4 count.

## Methods

This was a case control study done on HIV positive patients newly initiated on ART at KMC hospital, Mangalore. IRIS was diagnosed based on National Aids Control Organization Guidelines. Cases were defined as those who developed IRIS after initiation of ART and controls who did not develop IRIS.

## Results

40 cases and 80 controls were studied between May 2008 and May 2010, who were newly initiated on ART during this period. The cases and controls were compared based on age, sex, and initial CD4 count, final CD4 count, duration of ART, ART regimen, opportunistic infections and their relationship to CD4 count were analyzed. Mean age of patients who developed IRIS was 36 years. 75% of these patients were men and remaining 25% were females. Initial mean CD4 count was 135 and CD4 count at development of IRIS was 239. The mean duration of ART following which IRIS developed was 4 months. 65% of patients were on Lamivudine+Stavudine+Nevirapine regimen, 25% were on Zidovudine+Lamivudine+Nevirapine, 7.5% were on Stavudine+Lamivudine+Effavirenz, 2.5% were on Zidovudine+Lamivudine+Effavirenz.

Most common opportunistic infection occurring as IRIS was pulmonary tuberculosis followed by tubercular lymphadenitis and pneumocystis jheroveci pneumonia. Other opportunistic infections seen were oesophageal candidiasis, isospora duodenitis, CMV Retinitis, cryptococcal meningitis, herpes zoster. Tuberculosis accounted for 52.5% of cases of IRIS.

## Conclusion

Most common IRIS was tuberculosis followed by pneumocystis. Tuberculosis as IRIS occurred 2.5 months after initiation of ART. There was weak correlation between low CD4 count at ART initiation and occurance of IRIS.

